# Non-medical prescribing in the United Kingdom National Health Service: A systematic policy review

**DOI:** 10.1371/journal.pone.0214630

**Published:** 2019-07-29

**Authors:** Emma Graham-Clarke, Alison Rushton, Timothy Noblet, John Marriott

**Affiliations:** 1 School of Pharmacy, Institute of Clinical Sciences, College of Medical and Dental Sciences, University of Birmingham, Birmingham, United Kingdom; 2 Centre of Precision Rehabilitation for Spinal Pain, School of Sport, Exercise and Rehabilitation Sciences, College of Life and Environmental Sciences, University of Birmingham, Birmingham, United Kingdom; 3 Physiotherapy Department, St George's University Hospitals NHS Foundation Trust, London, United Kingdom; University of Malta Faculty of Health Sciences, MALTA

## Abstract

**Introduction:**

Non-medical prescribing was introduced into the United Kingdom (UK) to improve patient care, through extending healthcare professionals’ roles. More recent government health service policy focuses on the increased demand and the need for efficiency. This systematic policy review aimed to describe any changes in government policy position and the role that non-medical prescribing plays in healthcare provision.

**Method:**

The systematic policy review included policy and consultation documents that describe independent non-medical prescribing. A pre-defined protocol was registered with PROSPERO (CRD42015019786). Professional body websites, other relevant websites and the following databases were searched to identify relevant documents: HMIC, Lexis Nexis, UK Government Web Archive, UKOP, UK Parliamentary Papers and Web of Science. Documents published between 2006 and February 2018 were included.

**Results and discussion:**

Following exclusions, 45 documents were selected for review; 23 relating to policy or strategy and 22 to consultations. Of the former, 13/23 were published 2006–2010 and the remainder since 2013. Two main themes were identified: chronological aspects and healthcare provision. In the former, a publication gap for policy documents resulted from a change in government and associated major healthcare service reorganisation. In the later, the role of non-medical prescribing was found to have evolved to support efficient service delivery, and cost reduction. For many professions, prescribing appears embedded into practice; however, the pharmacy profession continues to produce policy documents, suggesting that prescribing is not yet perceived as normal practice.

**Conclusion:**

Prescribing appears to be more easily adopted into practice where it can form part of the overall care of the patient. Where new roles are required to be established, then prescribing takes longer to be universally adopted. While this review concerns policy and practice in the UK, the aspect of role adoption has wider potential implications.

## Introduction

Nurse prescribing was introduced in the United States of America in the 1960’s with gradual introduction into other countries since then [1]. In 2011 Kroezen et al [1] reviewed the nurse prescribing literature, identifying seven countries (Australia, Canada, Ireland, New Zealand, Sweden, United Kingdom, and United States of America) that had implemented nurse prescribing, with a further three countries where it was under consideration. They established that nurse prescribing was often subjected to a closed formulary or limited by the medical conditions treated, remaining subordinate to medical jurisdiction, with the United Kingdom (UK) and Ireland notable exceptions. Kroezen et al also commented that developments with regard to nurse prescribing were slow overall, apart from in the UK. Since that paper, the UK has pioneered the expansion of prescribing to other non-medical professions, providing a healthcare delivery model that could be utilised by other countries.

Traditionally, prescribing of human medicines had been perceived as a medical role, with only medical professionals and dentists having full prescribing rights in the UK. Two seminal reports challenged this view; the Cumberlege report [2] which paved the way for limited prescribing by health visitors and district nurses, and the Crown report [3], which recommended extending prescribing rights for the benefit of patients and to utilise the skills of healthcare professionals. The main UK healthcare provider, within which prescribers practice, is the National Health Service (NHS); established in 1948 to provide comprehensive healthcare to all, free at the point of delivery [4]. The UK also has a parallel smaller privately funded healthcare sector. Healthcare policy is directed by the UK government, reflecting the principles of the governing party at the time. Since 1948, this has been one of two main political parties (Labour, Conservative), apart from 2010–2015 when a Conservative and Liberal Democrat coalition was in power. As a general principle, Conservative governments tend to support free markets and expansion of the private sector, whereas Labour governments support the NHS over the private sector. Rising costs and changes in healthcare practice have led to numerous reforms since the NHS was founded but, irrespective of the political stance, the founding principles remain [4, 5].

In 2000 the governing Labour Party published a White Paper ‘The NHS Plan’, which described the government’s intention to modernise healthcare services, breaking down the traditional demarcations between professions and introducing new ways of working to increase healthcare capacity, shorten waiting times, and thus improve the patient experience [6]. Nurse prescribing was highlighted as one of the 10 key roles defined by the Chief Nursing Officer and the White Paper also included broad reference to ‘therapists’ (a generic term covering the professions allied to health) extending their roles, with prescribing included within this [6]. To support these sweeping changes to traditional practice the government established the Modernisation Agency, tasked with supporting service redesign at a local level [7], and launched a consultation on extending nurse prescribing [8]. This was followed in 2002 by a consultation on the introduction of supplementary prescribing for nurses and pharmacists [9], with approval granted later that year [10].

Supplementary prescribing is described as a voluntary partnership between the supplementary prescriber, the doctor looking after the patient, and the patient. The supplementary prescriber is then responsible for managing and prescribing the condition(s) and medication(s) listed in an agreed clinical management plan [11] but is unable to prescribe any other medication. The first supplementary nurse prescribers qualified in 2003, with pharmacists following in 2004. It quickly became apparent that supplementary prescribing, whilst ideal for complex and long-term conditions, had significant limitations with regard to acute care, hampering the government’s desire to enhance patient care through expanding nurse and pharmacist roles and hence improving access to medication. This was articulated clearly in the consultation documents launched in 2005 to investigate expansion into independent prescribing [12, 13]. Unlike supplementary prescribers, independent prescribers are accountable for the care of the patient, including examination and prescribing; Table 1 gives an overview comparing supplementary and independent prescribing. In addition, the British National Formulary provides an overview of independent non-medical prescribing, including the restrictions that the various professions must abide by (https://bnf.nice.org.uk/guidance/) [14]. All non-medical prescribers are required to complete a certified training course, be registered with their professional regulator, and to only prescribe within their professional expertise and competence.

**Table 1:** Comparison of independent and supplementary prescribing.

	Prescriber type	
	Independent	Supplementary
Accountable for care	√	X
Assess the patient	√	If required as part of the clinical management plan
Diagnose/confirm diagnosis	√	X
Plan clinical management	√	X
Prescribe	√	√
Range of medication	Any permitted by profession relevant legislation	Any medication or class of medication listed in the agreed clinical management plan and permitted by profession relevant legislation

Legislation to implement independent prescribing by nurses and pharmacists was enacted in 2006 [15], and since that time independent prescribing rights have been gradually extended to a range of healthcare professionals, most recently paramedics [16]. Although non-medical prescribing (NMP) is the umbrella term used to cover all prescribing by professions other than doctors, in this paper it refers to independent non-medical prescribing only.

This paper refers to the activities and qualifications of non-medical professionals in the UK. As these may vary internationally, a brief resume of the UK position is given in Table 2. Prescribing forms part of advanced clinical practice, a loose definition that Health Education England describes as involving making complex decisions at a high level of autonomy and encompassing four components: clinical expertise, leadership, education, and research [17].

**Table 2:** Brief resume of non-medical professions.

Profession	Initial qualification	Regulator	Medically qualified	Core activities	Advanced practice examples	Further details on scope of practice available from:
Diagnostic radiographer	BSc	HCPC	No	Conduct imaging tests on patients using ionising and non-ionising radiation. Use contrast agents or other medication where necessary for investigations	Interpretation and reporting on images Ultrasound guided biopsies	Society of Radiographers: https://www.sor.org
Nurse	BSc	NMC	No	Provide care for patients, assessing needs and delivering treatment plans	Work autonomously to manage a patient case load in a specialist area e.g. pain management Run nurse-led minor injury clinics	Royal College of Nursing: https://www.rcn.org.uk
Optometrist	BSc	GOC	No	Test sight and examine eyes. Prescribe lenses. Detect ocular disease and abnormalities.	Diagnose, assess and manage (including prescribing) ophthalmic conditions–for example glaucoma	The College of Optometrists: https://www.college-optometrists.org
Paramedic	Diploma, foundation degree, BSc, apprenticeship	HCPC	No	Assess, treat, stabilise and transfer patient to appropriate care centre	Diagnose and treat patients. Work in an urgent care centre, or GP practice to assess and treat patients	College of Paramedics: https://www.collegeofparamedics.co.uk
Pharmacist	MPharm	GPhC	No	Supply medicines to patients, ensuring that they are appropriate for the patient and of suitable quality. Provide medicines related advice	Work autonomously managing a patient case load in a specialist area e.g. renal failure, chronic pain Work in Emergency Departments to independently manage and treat patients.	General Pharmaceutical Council: https://www.pharmacyregulation.org Royal Pharmaceutical Society: https://www.rpharms.com
Physicians associate	Life sciences degree	No regulator Voluntary register held by Faculty of Physician Associates	No	Work alongside medical staff to care and treat patients *(the nearest USA equivalent role is physician’s assistant*.*)*	Not applicable	Faculty of Physician Associates: https://www.fparcp.co.uk
Physiotherapist	BSc or MSc	HCPC	No	Use various techniques to enable patients to improve movement and function and manage pain.	Work independently to manage a patient caseload in a specialist area e.g. back pain or respiratory failure Utilise techniques such as acupuncture, steroid injections or botulinum toxin injections	Chartered Society of Physiotherapy: https://www.csp.org.uk
Podiatrist	BSc	HCPC	No	Diagnose and treat common foot problems	Conduct podiatric surgery Specialise in areas e.g. diabetes care or sports medicine; utilising techniques such as acupuncture and steroid injections	The College of Podiatry: https://cop.org.uk
Therapeutic radiographer	BSc	HCPC	No	Use radiotherapy to treat cancer patients.	Plan radiotherapy treatment Independently manage and treat patients throughout the course of their radiotherapy	Society of Radiographers: https://www.sor.org

BSc–Bachelor of Science, MPharm–Master of Pharmacy, MSc–Master of Science, GOC–General Optical Council, GPhC–General Pharmaceutical Council, HCPC–Health and Care Professions Council, NMC–Nursing and Midwifery Council

The initial focus of government policy with regard to NMP was the desire to improve patient access to medicines. However, more recent documents from NHS England have focused on the increased demand for services and the need to drive efficiency so that maximum benefit can be obtained from the limited NHS budget [18, 19]. The role of NMP has been less apparent in these later documents, and it is unclear if this reflects a change in government policy.

The aim was to conduct a systematic policy review investigating changes in UK Government policy position with regard to NMP, since the introduction of independent prescribing for nurses and pharmacists. The review also aimed to determine the current role of independent NMP in the delivery of healthcare in the NHS, providing a snapshot of a dynamic situation.

## Method

### Protocol and registration

A systematic policy review was conducted to explore the evolution of government policy concerning independent NMP in the UK. To ensure transparency and enhance rigour a predefined protocol was developed in line with the PRISMA-P statement [20] and registered with PROSPERO (CRD42015019786) (S1 Protocol). The results are reported following the PRISMA statement (S1 Appendix) [21].

### Eligibility criteria

Documents describing policy concerning independent NMP in the UK were included. These included White and Green Papers, policy statements, consultation documents and reports. Documents published since 2006 were included, as the legislation permitting nurse and pharmacist independent prescribing was enacted in that year [15].

### Information sources

Advice was taken regarding appropriate electronic databases and websites to search (listed in Table 3) and to aid development of search strategies. Broad search terms (e.g. prescribing, non-medical) were used to capture as wide a range of documents as possible. Boolean operators and truncation were used if the database supported them. Iterative and ‘snowball’ search techniques were employed [22], with the primary searches complete to the end of February 2018, and secondary searches conducted as necessary (S2 Appendix). Documents obtained were mapped to identify gaps (for example, documents relating to the consultation process or profession specific policy documents) enabling targeted secondary searches to be conducted. Relevant citations in the reviewed documents were also obtained and personal files searched [22]. Full texts of the selected documents were screened to remove those that did not meet the eligibility criteria.

**Table 3:** Databases and websites searched.

Databases and websites	Professional body websites
Google Scholar	Chartered Society of Physiotherapists
HMIC—Ovid	College of Optometrists
Lexis Nexis	College of Paramedics
UK Government Web Archive	General Optical Council
UKOP (UK Official Publications)	General Pharmaceutical Council
UK Parliamentary Papers—ProQuest	Health and Care Professions Council
Web of Science	Institute of Radiology
www.gov.uk	Nursing and Midwifery Council
www.health-ni.gov.uk	Royal College of Nursing
www.publications.scot.nhs.uk	Royal Pharmaceutical Society
www.scot.nhs.uk	The Association of Ambulance Chief Executives
www.wales.nhs.uk	The College of Podiatry
	The Royal College of Radiologists

### Policy document selection

Two reviewers (EGC and TN) independently conducted each stage, resolving differences by discussion, with a third reviewer (AR) available if required for mediation [23]. Numbers excluded were recorded [21, 23].

### Data collection process and data items

Selected documents were entered into a Microsoft ® Excel for Mac (version 16) spreadsheet. Home nation and professions covered by the reference were noted, and whether the reference related to policy or consultation. The full texts were read, and notes made of any reference to NMP, including the context.

### Risk of bias assessment

Unlike research papers, whether qualitative or quantitative in nature, policy and consultation documents are not developed according to well-recognised principles. Risk of bias assessment is therefore not appropriate for this type of document and was not conducted. Policy documents are liable to be biased towards the ethos of the government in power at the time and documents produced by profession specific bodies towards their profession. The results are reported according to the relevant government era and, where appropriate, the specific professional body.

### Data syntheses

Information from the selected documents underwent narrative synthesis with visual depictions, described as an appropriate approach for non-research documents [24–26]. Following tabulation and data extraction, the selected documents were grouped depending on whether they concerned policy or consultation. To aid this process and to visualise the time distribution they were also plotted on a timeline, with a further timeline developed for the consultation documents. Using these techniques, a narrative summary was able to be developed by one researcher (EGC), and the findings were then debated and critically assessed by all authors to reach agreement.

One of the authors (EGC) is a practising pharmacist independent prescriber and NMP lead for an acute trust. In this role they support other non-medical prescribers and have an interest in NMP developments. This researcher standpoint is balanced by the other authors, who do not have prescribing qualifications.

## Results

### Policy document selection and characteristics

**Fig 1:**
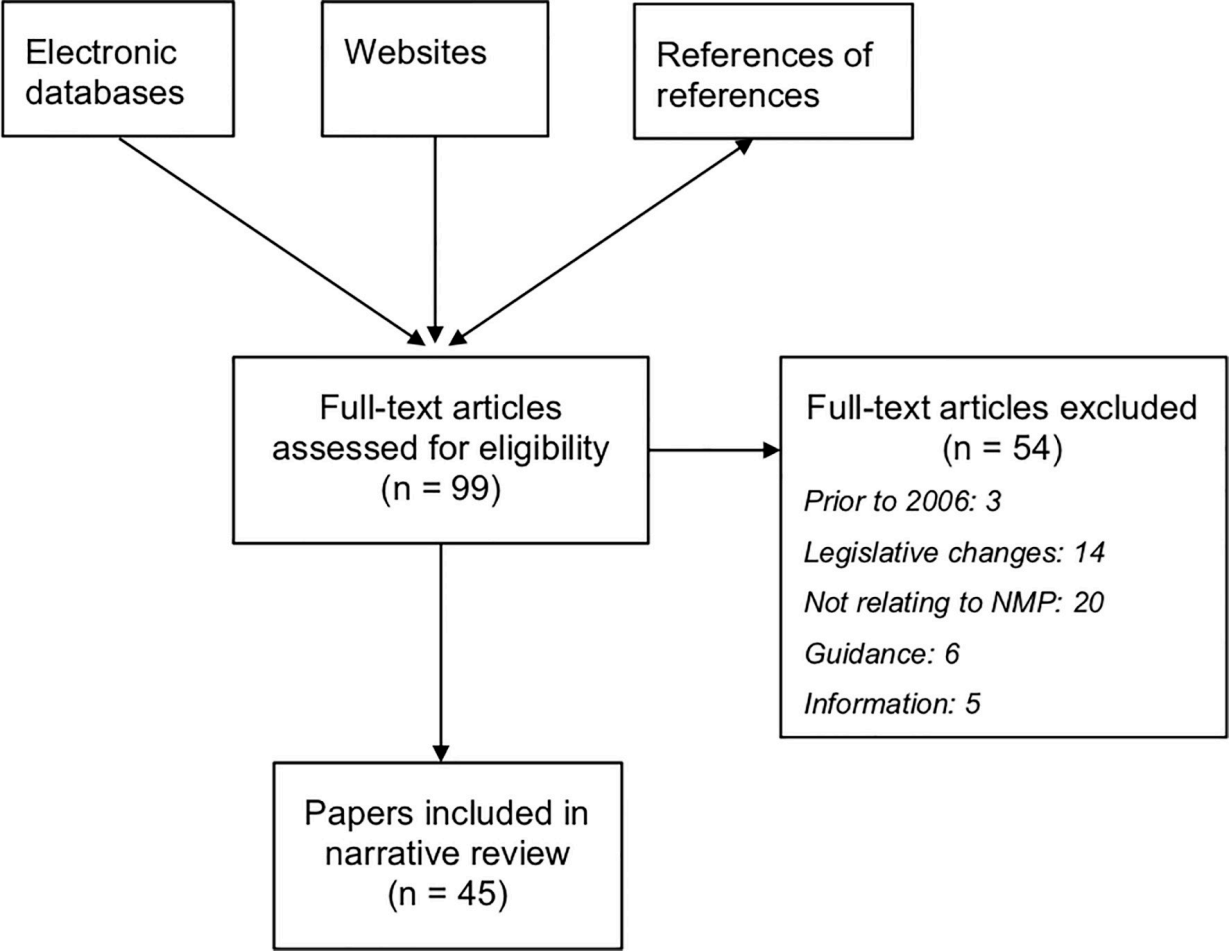
PRISMA paper selection flow diagram.

The search strategy identified 99 full text articles to be assessed for inclusion. Following exclusions, 45 documents were included in the review (Fig 1).

Of the included documents, 23 relate to policy or strategic report documents (see Table 4), and 22 to the consultation process concerning extension of independent NMP responsibilities to various healthcare professions (see Table 5).

**Table 4:** Policy and strategic report documents.

Title	Source	Date	Home Nation	Professional Group									Brief overview of contents
				Nurse	Pharmacist	Physiotherapist	Podiatrist	Paramedic	Radiographer	Optometrist	AHP	NMP	
**Improving Patients' Access to Medicines: A Guide to Implementing Nurse and Pharmacist Independent Prescribing within the NHS in England**	Department of Health	Apr-06	England	Y	Y								Highlights aims of independent prescribing Describes the scope of everything needed to implement independent prescribing
**Medicines Matters. A guide to mechanisms for the prescribing, supply and administration of medicines**	Department of Health	Jul-06	United Kingdom	Y	Y					Y			Describes the prescribing, supply and administration of medicines, Including the aims of the non-medical prescribing program
**Guidance for Nurse Independent Prescribers and for Community Practitioner Nurse Prescribers in Scotland: A Guide for Implementation**	Scottish Executive Health Department	Aug-06	Scotland	Y									Highlights aims of independent prescribing Describes the scope of everything needed to implement independent prescribing
**Improving Patients’ Access to Medicines: A Guide to Implementing Nurse and Pharmacist Independent Prescribing within the HPSS in Northern Ireland**	Department of Health Social Services and Public Safety	Dec-06	Northern Ireland	Y	Y								Highlights aims of independent prescribing Describes the scope of everything needed to implement independent prescribing
**The best medicine: the management of medicines in acute and specialist trusts**	Commission for Healthcare Audit and Inspection	Jan-07	England	Y	Y								Covers all aspects of medicines management in secondary care Includes a brief mention of non-medical prescribing
**Mental Health: New Ways of Working for Everyone. Progress Report**	Department of Health, National Institute for Mental Health in England National Workforce Programme	Apr-07	England	Y									Covers progress with developing New Ways of Working, and plans and strategies for further development. Described how non-medical prescribing will support these changes in practice
**Non medical prescribing in Wales—A guide for implementation**	Welsh Assembly Government	Jul-07	Wales	Y	Y								Highlights aims of independent prescribing Describes the scope of everything needed to implement independent prescribing
**New Ways of Working for Everyone: A best practice implementation guide**	Department of Health, National Institute for Mental Health in England National Workforce Programme	Oct-07	England									Y	Provides guidance on implementing New Ways of Working, using theoretical examples to illustrate points Examples include the use of non-medical prescribing
**Consultation on A Safe Prescription: Developing Nurse, Midwife and Allied Health Profession (NMAHP) Prescribing in NHS Scotland**	The Scottish Government, Primary Care Division	Nov-07	Scotland	Y							Y		Consultation strategy paper covering implementation of non-medical prescribing and the role of non-medical prescribing in service development and redesign
**Pharmacy in England: Building on strengths–delivering the future (Cm 7341)**	Department of Health	Apr-08	England		Y								Government White Paper describing the current role of pharmacy and how pharmacy skills could be better utilised Includes use of prescribing by pharmacists with case studies as examples
**Allied health professions prescribing and medicines supply mechanisms scoping project report**	Department of Health	Jul-09	England			Y	Y		Y				Describes current position with regard to AHPs and their changing role Highlights that expansion of prescribing rights would improve patient care, with examples Identifies priorities in prescribing expansion
**A safe prescription; Developing nurse, midwife and allied health profession (NMAHP) prescribing in NHS Scotland**	The Scottish Government	Sep-09	Scotland	Y							Y		Final version of the consultation strategy paper Includes key healthcare policy drivers where non-medical prescribing may be beneficial
**Pharmacist Prescriber Training Working Group Report for the MPC Programme Board**	Medical Education England	Jan-10	England		Y								Describes the background to the pharmacist prescribing, current context and future developments Highlights changes to undergraduate teaching that should occur to optimise pharmacist as prescribers
**Prescription for Excellence**	The Scottish Government	Sep-13	Scotland		Y								Describes the Scottish vision that all pharmacists will become independent prescribers, working in partnership with medical practitioners
**Now or never: shaping pharmacy for the future**	The Royal Pharmaceutical Society	Nov-13	England		Y								Covers the current pharmacy activity and potential future developments. Include examples of pharmacist prescribers and mentions how many have qualified. Highlights poor awareness of pharmacy profession by patients and wider healthcare service
**Seven Day Services in Hospital Pharmacy: Giving patients the care they deserve**	The Royal Pharmaceutical Society	Jun-14	United Kingdom		Y								Describe the challenges in moving to full seven-day services Gives examples of pharmacist prescribers supporting seven-day services
**Our Plan for Primary Care in Wales up to March 2018**	Welsh Assembly, NHS Wales	Nov-14	Wales	Y	Y			Y					Highlights general practice doctors’ workforce shortfall Highlights how healthcare professionals can support general practitioners, including non-medical prescribing
**A Planned Primary Care Workforce for Wales: Approach and development actions to be taken in support of the plan for a primary care service in Wales up to 2018**	Welsh Assembly, NHS Wales	Jun-15	Wales	Y	Y	Y				Y			Covers workforce development, profession by profession, to enable support for general practitioners Highlights the need for expansion in non-medical prescribers
**The future of primary care: Creating teams for tomorrow**	Health Education England	Jul-15	United Kingdom	Y	Y	Y							Describes the challenges in general practice Highlights development of non-medical professionals to support general practice
**Transformation of seven day clinical pharmacy services in acute hospitals**	NHS England	Sep-16	England		Y								Describes the actions needed to develop seven day working Includes examples of pharmacist prescribing supporting the multi professional team
**Improving care for people with Long Term Conditions**	The Royal Pharmaceutical Society	Nov-16	England		Y								Describes improving care of patients with long term conditions, utilising pharmacists’ skills Recommends prescribing as a key skill
**The General Practice Nursing Workforce Development Plan**	Health Education England	Mar-17	England	Y									Review of general practice nursing, highlighting practice role and potential workforce issues Identifies challenge of freeing time for prescribing training
**Facing the Facts, Shaping the Future: A draft health and care workforce strategy for England to 2027**	Public Health England	Dec-17	England	Y	Y								Describes the current workforce issues including recruitment and retention Reviews this in context of services and of individual staff groups

AHP–allied health professional, NMP–Non-medical prescriber

**Table 5:** Consultation documents.

Title	Source	Date	Home Nation	Professional Group							Brief overview of contents
				Nurse	Pharmacist	Physiotherapist	Podiatrist	Paramedic	Radiographer	Optometrist	
**Consultation on proposals to introduce independent prescribing by optometrists (MLX 334)**	Medicines & Healthcare Products Regulatory Agency	Aug-06	United Kingdom							Y	Describes scenarios where optometrist prescribing would be beneficial Includes options for immediate referral and management of long-term conditions
**Public consultation—independent prescribing of controlled drugs by nurse and pharmacist independent prescribers (MLX338)**	Home Office, Drug Strategy Unit	Mar-07	United Kingdom	Y	Y						Includes risk and impact assessments Highlights that controlled drug prescribing would support the aims of improving patient care and choice
**Public consultation (MLX 334): Proposals to introduce independent prescribing by optometrists—outcome**	Medicines & Healthcare Products Regulatory Agency	Aug-08	United Kingdom							Y	Report of outcome of public consultation, including confirmation that CHM recommend optometrist prescribing
**Proposals to introduce prescribing responsibilities for paramedics: stakeholder engagement**	Department of Health	Mar-10	United Kingdom					Y			Highlights scenarios where prescribing would be beneficial Discusses which paramedics would be suitable, and planned safeguards
**Engagement exercise: To seek views on possibilities for introducing independent prescribing responsibilities for podiatrists**	Department of Health	Sep-10	United Kingdom				Y				Described, with examples, podiatry roles and training Describes potential benefits of independent prescribing Uses open questions to gain information from stakeholders
**Engagement exercise: To seek views on possibilities for introducing independent prescribing responsibilities for physiotherapists**	Department of Health	Sep-10	United Kingdom			Y					Described, with examples, physiotherapy roles and training Describes potential benefits of independent prescribing Uses open questions to gain information from stakeholders
**Proposals to introduce independent prescribing by podiatrists: impact assessment**	Department of Health	Jul-11	United Kingdom				Y				Describes potential financial and other benefits from streamlined pathways for each option under consideration
**Consultation on proposals to introduce independent prescribing by podiatrists**	Department of Health	Sep-11	United Kingdom				Y				Public consultation describing current role of podiatrists and scenarios where prescribing would be beneficial Seeks clarification on areas such as education and governance Prescribing unlicensed medication excluded following engagement exercise
**Consultation on proposals to introduce independent prescribing by physiotherapists**	Department of Health	Sep-11	United Kingdom			Y					Public consultation describing current role of physiotherapists and scenarios where prescribing would be beneficial Seeks clarification on areas such as education and governance Prescribing unlicensed medication excluded following engagement exercise
**Summary of the Commission on Human Medicines meeting held on Thursday 17th & Friday 18th May 2012**	Commission on Human Medicines	May-12	United Kingdom			Y	Y				Reports that the committee was able to support independent prescribing for podiatrists and physiotherapists in line with results from consultation exercise
**Summary of Public Consultation on Proposals to Introduce Independent Prescribing by Physiotherapists**	Department of Health	Jul-12	United Kingdom			Y					Majority of respondents supported independent prescribing from a full formulary There was also support for a limited list of controlled drugs and to allow mixing of medicines
**Proposals to introduce independent prescribing by physiotherapists: impact assessment**	Department of Health	Jul-12	United Kingdom			Y					Describes potential financial and other benefits from streamlined pathways for each option under consideration Includes risk and governance
**Summary of Public Consultation on Proposals to Introduce Independent Prescribing by Podiatrists**	Department of Health	Jul-12	United Kingdom				Y				Majority of respondents supported independent prescribing from a full formulary There was also support for a limited list of controlled drugs and to allow mixing of medicine
**Independent prescribing by radiographers: Impact Assessment**	NHS England	Jan-15	United Kingdom						Y		Set out a policy background and describes scenarios where prescribing may be beneficial e.g. managing radiotherapy side effects Describes financial costs, governance arrangements and potential risks
**Consultation on proposals to introduce independent prescribing by radiographers across the United Kingdom**	NHS England	Feb-15	United Kingdom						Y		Public consultation Describes current role and scenarios where prescribing may be beneficial Describes governance proposals
**Consultation on proposals to introduce independent prescribing by paramedics across the United Kingdom**	NHS England	Feb-15	United Kingdom					Y			Public consultation Describes paramedic roles and changes in practice that result in more patients being treated at home Highlights that this would be for advanced paramedics
**Proposal to introduce independent prescribing by paramedics: impact assessment**	NHS England	Feb-15	United Kingdom					Y			Highlights current issues and rationale for prescribing Details of the various options and associated costs Identifies potential risks
**Commission on Human Medicines and Expert Advisory Group Final Summary Minutes**	Commission on Human Medicines	Oct-15	United Kingdom					Y	Y		Describes that committee was unable to support paramedic or diagnostic radiographer independent prescribing The Committee was able to support the therapeutic radiographer independent prescribing
**Independent prescribing by therapeutic radiographers**	NHS England	Jan-16	United Kingdom						Y		Impact assessment for therapeutic radiographers only Set out policy background and describes scenarios where prescribing may be beneficial Describes financial costs, governance arrangements and potential risks
**Summary of the responses to the public consultation on proposals to introduce independent prescribing by paramedics across the United Kingdom**	NHS England	Feb-16	United Kingdom					Y			Majority of respondents supported independent prescribing by paramedics There was also support for a limited list of controlled drugs and to allow mixing of medicine
**Summary of the responses to the public consultation on proposals to introduce independent prescribing by radiographers across the United Kingdom**	NHS England	Feb-16	United Kingdom						Y		Majority of respondents supported independent prescribing from a full formulary There was also support for a limited list of controlled drugs and to allow mixing of medicines It was noted that the CHM supported independent prescribing for therapeutic radiographers only
**Summary of the Commission on Human Medicines meeting held on Thursday 7th September 2017**	Commission on Human Medicines	Sep-17	United Kingdom					Y			Brief notes that feedback on independent prescribing by paramedics had been considered and discussed, and that they would now endorse the recommendation to support prescribing

CHM—Commission on Human Medicines

**Fig 2:**
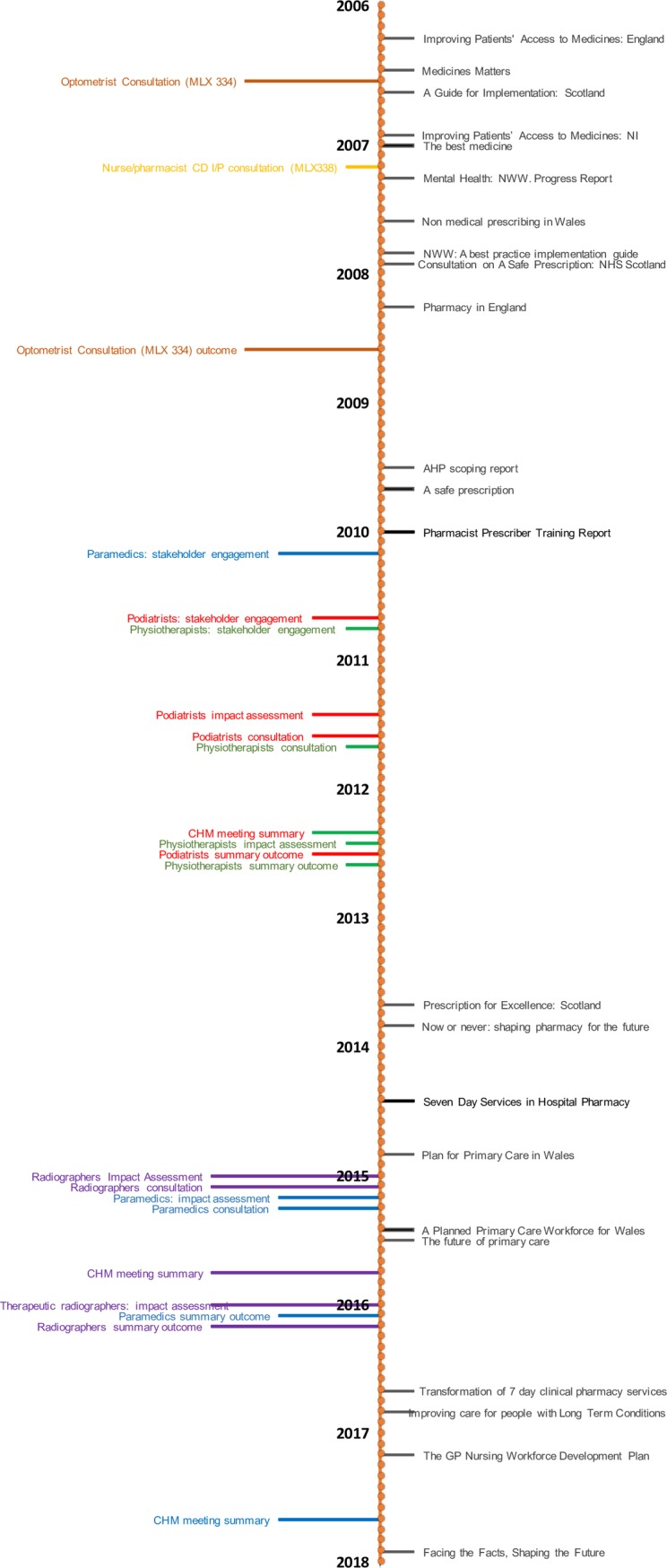
Timeline of selected documents. Policy–black, Optometrist–brown, Radiographer–purple, Nurse/Pharmacist–yellow, Paramedic–blue, Podiatrist–red, Physiotherapist- green.

The policy and strategic report documents relate to a single profession (nursing 3, pharmacy 7), multiple professions (12), or generic NMP (one). The majority concern matters in the home nations (England 12, Scotland 4, Wales 3 and Northern Ireland 1) with only 3 concerning the United Kingdom. They can be divided into two chronological eras, with just over half published between 2006 and 2010, and the remainder published since 2013 (Fig 2).

### Synthesis of results

#### The Labour Government era 2006–2010

Four of the early documents comprised guidance issued by the home nations to support NMP. These were released as the relevant regulations governing prescribing were amended to permit independent NMP. The first was released by the Department of Health in April 2006, coinciding with the initial changes in legislation and regulations permitting independent prescribing by nurses and pharmacists [15, 27, 28]. This was followed by Scotland’s guidance, released in July 2006, Northern Ireland’s guidance in December 2006 and the Welsh guidance in 2007 [29–31]. All four documents are similar in nature; however, Scotland’s relates to nurse prescribing only whereas the other three relate to nurse and pharmacist prescribing. This reflects the changes made by the home nations whereby England, Wales and Northern Ireland each introduced nurse and pharmacist independent prescribing simultaneously, whereas Scotland introduced nurse independent prescribing first, followed a year later by pharmacist independent prescribing. Although the bulk of these documents relates to practical implementation guidance, each states the core policy drivers behind NMP which were:

- improving patient care, without reducing safety
- making it easier to patients to access the medicines they require
- increasing patient choice
- utilising the skills of health professionals
- supporting team working

The Welsh guidance included the additional benefits of improving healthcare capacity and enhancing patient access for advice and services.

Scotland conducted a prescribing strategy consultation exercise, with the final strategy launched in 2009 [32, 33]. These documents covered independent prescribing by nurses and midwives and supplementary prescribing by allied health professionals but not pharmacist prescribers. They highlighted the variable adoption of NMP across Scotland and had the aim of improving uptake of NMP to support the NHS boards in delivering patient centred care.

There were two remaining prescribing specific documents in this era; the scoping report on Allied Health Professional (AHP) prescribing and a report on pharmacist prescribing training [34, 35]. The former reviewed the developing role of AHPs (see Table 2) and highlighted some of the limitations resulting from their inability to prescribe; identifying which professions would benefit most from the ability to prescribe, either independently or as a supplementary prescriber, and also which professions should not become prescribers. Additionally, the professions were prioritised, with physiotherapy and podiatry identified as high priorities for independent prescribing, followed by radiography. The latter document reviewed pharmacist prescribing experiences and recommended several changes to training, both at undergraduate level and regarding the prescribing course.

The remaining documents produced in this era, although generic in nature, include references to NMP. The first was a Department of Health document released in 2006 providing further guidance on medicine supply and reiterating the drive behind NMP [36]. The document included several proposed next stages for NMP:

- To consult on optometrist independent prescribing
- To promote nurse and pharmacist independent prescribing
- To review the prescribing needs of emerging roles

This was followed by the Audit Commission report in 2007 on medicines management, which mentioned the development of nurse and pharmacist prescribing and described the distribution of prescribers at that time [37]. Data collection had been in 2005 and 2006 and therefore the majority of these data would have been collected from supplementary prescribers. They recommended that trusts identify where NMP would provide the maximum benefit clinically and that work should be performed to identify why some non-medical prescribers did not prescribe regularly.

The “New Ways of Working in Mental Health” project released two documents in 2007, a progress report and an implementation guide [38, 39]. The progress report reiterated the five core drivers behind NMP and described how NMP should be incorporated into the changes in working practice such as multidisciplinary team working. The implementation guide provided theoretical examples of changed practice which incorporated NMP.

The final document in this era was the pharmacy White Paper [40]. This highlighted the roles that pharmacists could play in improving the healthcare of patients, including the example of prescribing in long-term conditions. Although some case studies were described, most of the suggested roles for prescribers were aspirational.

#### The Coalition and Conservative Governments era 2013–2017

The first two documents in this era both concerned the role of pharmacy in providing patient centred health care. The first of these was the Scottish Government’s vision for pharmacy which envisaged integration of pharmacists into all aspects of healthcare [41]. Central to this vision was the aim of having all pharmacists qualified as independent prescribers. The second document was a report by the Royal Pharmaceutical Society on pharmacy activity and future potential [42]. Various examples of prescribing practice are described (for example, pharmacists running cardiovascular and chronic pain clinics) but the comment is made that it is not sufficient simply to provide prescribing courses, that roles must also be developed that utilise this activity. The report contrasts the English and Scottish governments approach to pharmacy, to the detriment of the English government’s approach.

There are three further pharmacy specific documents in this era, with two of these concerning seven-day hospital clinical pharmacy services. The first was a report by the Royal Pharmaceutical Society discussing potential approaches to providing a seven-day service and the associated challenges [43]. Examples where seven-day pharmacy services had been implemented were given, with many of the contributors anticipating the use of pharmacist prescribers to support delivery. The second report, from NHS England, describes the need to deliver clinical pharmacy services seven days a week, highlighting the impact that pharmacy services make and describing the importance of prescribing to support the multi-professional team [44]. The final pharmacy specific document was the Royal Pharmaceutical Society produced policy, concerning care for patients with long-term conditions [45]. This highlights the role that pharmacists can play in supporting these patients, and makes a number of key recommendations, the first of which is that pharmacists should have the opportunity to become prescribers enabling them to manage treatment of these patients.

The Welsh Assembly produced a plan for primary care in 2014, followed by a primary care workforce development plan in 2015 [46, 47]. The first of these documents highlighted the increasing pressure on general practice from a combination of increasing demand, a shortage of general practitioners and financial constraints. The focus was on health rather than ill-health and to provide person centred care within the local community, using the most appropriate healthcare professional for the task. Advanced practice such as NMP was seen to relieve pressure on general practitioners. The associated workforce plan described the potential role of NMP for various professions and provided examples. One such example is the monitoring of low risk glaucoma patients by optometrists, and the document comments that there will be an increased need for optometrists to train as prescribers as they develop these advanced roles.

A report commissioned by Health Education England on primary care, published in 2015, described how primary care could be delivered using a wide range of healthcare professionals [48]. Included in the recommendations was the role of the prescribing pharmacist to support medicines optimisation activities, such as changing the medication of patients at risk of polypharmacy and adverse drug events, and the potential for physiotherapist prescribers to enable them to provide streamlined care for patients [48]. This was followed in 2017 by the general practice nursing workforce plan [49]. Prescribing is described as complementing the nursing role, but challenges are acknowledged particularly in enabling time for training. Finally in this era, there was the draft workforce strategy for England which was released for consultation in December 2017 [50]. This specifically mentioned prescribing in the pharmacy section, describing a project to put advanced pharmacists with prescribing qualifications into emergency departments, and also commented that increased numbers of nurse prescribers would be required in the community and primary-care sectors. No mention was made of prescribing by any other non-medical healthcare professional.

#### Consultation documents

Two public consultations, to gauge opinion, were launched during the period 2006–2008; the first concerned the introduction of independent prescribing for optometrists, and the second regarding controlled drug prescribing by nurse and pharmacist independent prescribers. The consultation process for the introduction of independent prescribing by optometrists was launched in August 2006, with the outcome announced in 2008, and associated legislation passed the same year [51–53]. This time period contrasts with the second consultation in 2007 on controlled drug prescribing, where agreement that this should be permitted was reached, but changes in legislation were not enacted until 2012 [54–56].

**Fig 3:**
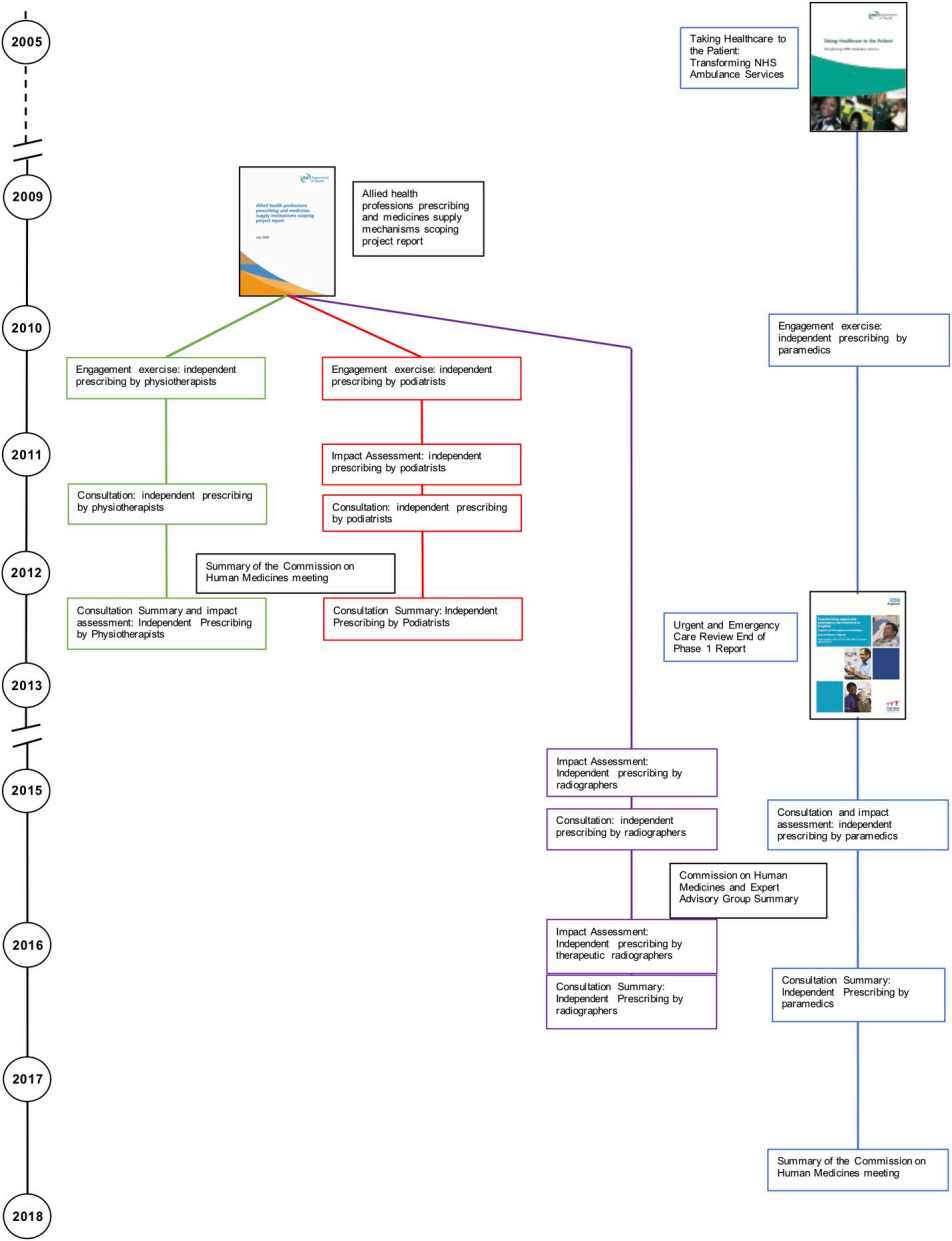
Consultation timeline.

Following the 2009 AHP scoping report, stakeholder engagement exercises were launched in 2010 to investigate independent prescribing rights for both podiatry and physiotherapy, followed by consultation exercises in 2011 and the outcome and approval in 2012, the whole process taking a little under two years [57–65]. The consultation for radiographers was launched in 2015 with approval for therapeutic radiographers only granted in 2016 (diagnostic radiographers were excluded) [66–70]. These relatively short consultation exercises contrast strongly with that of the paramedics. The initial document mentioning paramedic prescribing had been published in 2005 [71], with the stakeholder engagement exercise held in 2010, a year before that of the podiatrists and physiotherapists [57, 61, 72]. The potential for paramedic prescribing was reiterated in the 2013 urgent care report, which described the changing role of paramedics, and the potential for further role extension such as treatment at home by a paramedic to reduce demand on emergency care services [73]. Furthermore, when the formal paramedic consultation process began, advanced paramedics had started to work in a range of settings such as emergency care departments as well as the more traditional ambulance service (see Table 2) [74]. The paramedic and radiographer consultation exercises ran simultaneously, but final approval for paramedics was only granted in 2017 [74–78]. A comment is made in the related paramedic impact assessment that the consultation exercise was delayed because of capacity issues [75]. The relative timescales are visually depicted in Fig 3.

## Discussion

### Summary of evidence

This is the first such policy review bringing together the UK policy documents concerning NMP to describe the role of this evolving activity. The document review reveals two main themes, which are expanded on below. The first theme highlights issues arising from inspecting the chronological aspects of the selected documents. The second theme covers the evolving approach to healthcare provision and describes how NMP has become embedded into routine practice for many non-medical prescribers. However, differences in practice remain and these are highlighted.

#### Chronological aspects

Inspection of the timeline of included documents reveals a noticeable gap between 2010 and 2013, when no reports or strategic documents concerning NMP were released by a government body. The beginning of this period coincides with the change in government in 2010 from Labour to the Coalition. Two factors are likely to be responsible for this dearth of publications. Firstly, the Coalition embarked on an overall reorganisation of the NHS in England, initiated in the 2010 White Paper ‘Equity and Excellence’, and enacted through the Health and Social Care Act in 2012 [79, 80]; focussing on the high level structure rather than finer detail. Secondly, the country had been in economic recession since 2008 and the Coalition’s 2010 budget introduced austerity measures designed to reduce the nation’s budget deficit and improve economic growth [81, 82]. The government attempted to protect the NHS from financial cuts implemented more generally across all services, however the funding growth rate for the NHS in England was curtailed to 1.4% a year compared with 6% a year under the previous Labour government [83]. Government priorities were therefore concerned with major reform of the NHS structure and introduction of commissioning groups, rather than the continued development of existing practices.

The change in government also probably explains the delay in extending controlled drug prescribing for nurses and pharmacist independent prescribers. Extending controlled drug prescribing rights requires the agreement of the Department of Health, the Home Office, the Medicines and Healthcare Products Regulatory Agency and the Advisory Council on the Misuse of Drugs (ACMD), and, subsequently, amendments to the Misuse of Drugs Regulations 2001 and medicines legislation [54]. The consultation closed in June 2007, and in November 2007 the ACMD wrote to the Under-Secretary of State at the Home Office, and the Minister of State for Public Health at the Department of Health, to support the proposals and the change in legislation [84]. However, the required change in legislation was only enacted in 2012, and it can be surmised that with the Coalition’s priorities focused on reorganisation of the whole NHS, extending controlled drug prescribing to nurse and pharmacist independent prescribers was accorded low priority [55, 56].

The consultation processes for the AHPs (physiotherapists, podiatrists and radiographers) were all concluded within a reasonable timeframe, despite the change in government occurring between publication of the AHP scoping report and initiation of the physiotherapy and podiatry consultation exercises [34, 57, 61]. The AHP scoping report had demonstrated a clear role for prescribing for each of these professions in streamlining and improving patient care. In addition, the report prioritised which professions should be considered first, taking into consideration the strength of case for prescribing for each profession and the capacity of the Department of Health, and Medicines and Healthcare Products Regulatory Agency to conduct the necessary consultations. As an aside, the consultation exercises reflect the NHS reorganisation, with the physiotherapy and podiatry consultation exercises conducted under the auspices of the Department of Health, and subsequent consultation exercises under NHS England.

In comparison, the lack of clarity concerning how prescribing would be utilised by paramedics, and their evolving role, explains the extended time period between the initial recommendation regarding paramedic independent prescribing and final approval. At the time of the initial report paramedics had recently become registered with the Health Care Professions Council, and the NHS advanced practice role was developing [71] with a shift in training from resuscitation, to assessing and treating the patient at home. The urgent care report in 2013 highlighted the potential for treatment by paramedics to reduce demand on emergency care services [73]. Following the consultation, the Commission on Human Medicines (CHM) was unable to recommend prescribing by paramedics because of concern that paramedics would need training in a large range of conditions to ensure patient safety [66]. The minutes for the 2017 CHM meeting simply say that they endorse the recommendations for independent prescribing for paramedics, and it is to be presumed that they had been provided with reassurance concerning the training and role of paramedics [78].

#### Healthcare provision—evolution of policy

The five drivers for prescribing documented in the implementation guidance reiterated the aims of the 2000 NHS White Paper to improve patient care and break down the traditional demarcations between professions [6, 27, 29–31]. These and other early documents such as Medicines Matters, and the “Mental Health New Ways of Working” project were published before full independent prescribing was embedded [36, 38, 39]. As such, they discuss the potential for NMP to improve patient care and, in particular with the mental health documents, develop novel ways of working. Medicines Matters explicitly commented that NMP was unsuitable for patients with complex conditions, recommending the use of supplementary prescribing instead [36]. The pharmacy White Paper listed prescribing as one of the activities that pharmacists could undertake, including in the care of long-term conditions, but many of the examples are theoretical [40]. The 2009 AHP scoping report highlights the changing role of, for example physiotherapists or podiatrists, commenting that they may now be responsible for a full package of patient care but were hampered by the inability to prescribe independently [34]. Again, this document describes potential or theoretical benefits.

However, when the Scottish government published their NMP strategy, they were able to draw on a number of published papers providing evidence of the benefits [33], although in reality the only full independent prescribers included were nurses. Likewise the pharmacist prescriber training report in 2010 was also able to draw on practice examples to illustrate various different ways that independent prescribing had been implemented [35].

The 2010 White Paper ‘Equity and excellence: liberating the NHS’ signalled a change in direction for the health service, putting the patient at the centre of care with ‘*no decision about me without me*’ [79] but without the previous emphasis on workforce development; a point highlighted in a later staffing report [85]. The need for responsive and patient centred care, within the constraints of limited finances, was further developed in the subsequent Five-Year Forward View [19]. This document sets the need to provide more integrated care, giving patients greater control, against the background of increasing demand, rising costs resulting from new technologies, and budgetary constraints. Although prescribing is not specifically mentioned, there is a call to challenge traditional ways of working and to use the most appropriate healthcare professional for the task in hand.

This approach is echoed by the Welsh Assembly primary care plan, which describes a future model of primary-care in which the general practitioner acts as the leader over a multi professional team, who between them care for the patient [46]. The Welsh Assembly associated workforce development plan depends on other healthcare professionals taking on roles traditionally associated with general practitioners or secondary care, with NMP perceived as integral to these developments [47]. The English primary care report [48] describes a number of approaches to reducing the burden on general practitioners. Included in this are new models of practice such as the work of physicians’ associates (see Table 2), but as The Health Foundation comments, their role in relieving pressure on doctors will be limited if they cannot prescribe [85]. Nurse prescribing is not specifically mentioned, although the report does identify that nurses have many responsibilities, including the care of patients with long-term conditions. More recently, the draft workforce strategy describes advanced practice for a number of professions such as nursing and paramedics but does not define what this entails [50]. It also describes podiatry and physiotherapy being potential first contact points for patients with musculoskeletal disorders. Prescribing would support all of these activities but is not explicitly mentioned and it could be perceived that NMP is seen to be so routine and embedded in practice for these professions that it warrants no mention. This compares with the pharmacy situation, where the same document put pharmacist independent prescribing as one of the priority areas to address. Other reports also make explicit mention of pharmacy prescribing as one of the tools to enhance medicines optimisation practices [44, 45] suggesting that pharmacist prescribing is still not embedded into routine practice.

A review of the professional distribution of policy documents supports this supposition concerning NMP becoming routine practice, with the majority involving generic NMP or covering multiple NMP professions (see Table 4). Of the three nursing specific policy documents, two date from before 2010, and the final one from 2017 [29, 38, 49]. Pharmacy alone of the professions is associated with multiple policy documents since 2013; with three by the Royal Pharmaceutical Society and one by each of the Scottish government and NHS England [41–45]. Similar recent policy documents were unable to be identified for any other of the NMP professions, despite in-depth searching. This may reflect the need for pharmacists to develop new roles and skills as the traditional dispensing role diminishes as a consequence of technological advances such as electronic prescribing and robotic dispensing. With medicines central to pharmacy practice, it is appropriate that these roles support medicines optimisation; however, these are not existing roles that a pharmacist can move into, rather they are roles that require creating. It is also notable that while community (drugstore) pharmacists comprise the majority of the profession, most prescribers are found in primary and secondary care instead, indicating challenges with adopting prescribing in community practice [86, 87]. The pharmacy orientated policy documents describe to both pharmacists and commissioners how pharmacist prescribing could work in practice. This compares with other healthcare professions, such as physiotherapy, where medicines form an adjunct to their main practice area, enhancing role expansion. Pharmacy could also be perceived to be an innately cautious profession [88], and the policy documents could thus serve to overcome a reluctance to adopt innovative working practices.

It is notable that there has been a shift regarding the role that NMP plays in the care of patients. The 2006 document, Medicines Matters, envisaged independent prescribers utilising a comparatively small personal formulary of drugs, excluding controlled drugs and unlicensed medicines, to treat uncomplicated conditions [36]. Since independent prescribing for nurses and pharmacists was launched, their prescribing rights have been gradually extended to include unlicensed medicines and controlled drugs [54, 89] and more recent documents describe the role NMP has in the care of long-term conditions and complex patients, such as palliative care [45, 48]. This is echoed by the changing role of medical staff in patient care. The early implementation guidance described medical staff retaining an overview of patient care, with nurse and pharmacist prescribing intended to improve patients’ access to medicines [27, 29–31]. Subsequent consultation processes (podiatry, physiotherapy, radiography and paramedics) have seen a change so that the examples given in these documents describe the provision of a complete package of care without the need to involve other healthcare professionals. Indeed, the consequent reduction in costs through reducing appointments is listed as a benefit in the impact assessments [60, 62, 68, 75]. More recently, the Health Education England primary care report envisages that general practitioners will be treating patients with complex conditions, with other healthcare professionals providing routine care [48].

### Strengths and limitations

The strengths of the present policy review include the systematic, iterative approach to identifying relevant policy documents, using document mapping techniques to identify missing documents. The dynamic nature of this healthcare area inevitably means that this review provides a snapshot of the situation between 2006 and 2018, which may well be superseded, for example if political changes resulting from unanticipated developments such as ‘snap’ general elections and referenda occur. The selected documents relate to the UK and the home nations only and this may limit generalisability to other countries. Additionally, although the legislation permits the use of NMP in UK private healthcare, the policy documents concern the use of NMP in the NHS and this may further limit generalisability for alternative healthcare systems. However, it can also be argued that the development in the UK could provide a roadmap for other countries wishing to expand their non-medical prescribing workforce, by providing examples of successful NMP implementation into routine practice.

Despite extensive searches there may well be further policy documents available, such as from the home nations or professional bodies that are not identifiable through a search strategy.

## Conclusions

In conclusion it can be seen that this policy review has revealed that the government approach to NMP has changed over the 12-year period from 2006. NMP was originally intended as a means of improving patient choice and access to medicines, whilst also developing the workforce. A subsequent change in government (and associated political ideology) combined with financial and staffing shortfalls have resulted in the emphasis subtly changing to NMP supporting, or even replacing, medical practitioners. Patients are expected to be cared for, and treated by, the most appropriate health care professional such as a physiotherapist for a musculoskeletal problem. Medical workload is thus reduced, enabling the more complex cases to still be treated by medical practitioners despite a reduction in their numbers. Costs are reduced by streamlining care through reducing multiple appointments with different healthcare professionals, and by using the most appropriately qualified professional.

This policy review has also highlighted the role that NMP now plays in patient care, with prescribing perceived as one activity in the advanced practice armamentarium used to treat and support patients, enabling patients to benefit from receiving a complete package of care from a single healthcare professional. As prescribing has become embedded into day to day practice for the majority of the NMP professions, so the need to highlight prescribing in policy documents has diminished (as seen in the recent workforce development document), just as it is no longer felt necessary to describe in detail advanced practice in these professions. As new models of practice are developed, such as use of physician’s associates, so the demand for NMP to expand to other healthcare professional groups continues, with the implication that prescribing is integral to these roles.

However, this policy review has found that while NMP has become embedded into routine practice for many professions, this is not universal. Despite pharmacists having achieved independent prescribing rights in 2006, it would appear from the repeated policy documents describing the need for pharmacist prescribers that it is still not embedded into pharmacists’ routine practice. Medicines remain at the core of pharmacy practice through supply and optimisation but, until the new roles become established, prescribing has yet to be perceived as a ‘normal’ pharmacist activity.

This policy review has also highlighted the practical impact that a change in government can have, as shown by the gap in policy document publication during the Coalition’s review and reorganisation of the NHS, and the delays in legislation concerning controlled drugs. However, these delays are not inevitable, as shown by the physiotherapist and podiatrist consultations which were conducted during this period.

While these findings concern a publicly funded health service in a single country, and may therefore be considered to have limited generalisability, there are messages that may resonate in other settings. These concern the impact of reorganisation on service development and how uptake of a novel activity is adopted by professions.

## Supporting information

S1 Appendix PRISMA checklist. (DOC)

S2 Appendix HMIC (Ovid) search strategy. (DOCX)

S1 Protocol PROSPERO record. (PDF)
