# Supplementary material for: Non-medical prescribing in the United Kingdom National Health Service: A systematic policy review
Source: PLoS One. 2019 Jul 29;14(7):e0214630. doi: 10.1371/journal.pone.0214630 (PMC6663007; doi:10.1371/journal.pone.0214630)
Supplement: S2 Appendix — (DOCX) [file pone.0214630.s002.docx]

**S2 Appendix. HMIC (Ovid) search strategy**

| 1. allied health professionals/ or physiotherapists/ or podiatrists/ or radiographers/ or allied health professions/ |  |
| --- | --- |
| 2. exp Prescribing/ |  |
| 3. exp Nurses/ |  |
| 4. exp Pharmacists/ |  |
| 5. exp Optometrists/ |  |
| 6. exp Paramedics/ |  |
| 7. 1 or 3 or 4 or 5 or 6 |  |
| 8. 7 and 2 |  |
| 9. 8 |  |
| 10. limit 9 to yr="2006 - 2018" |  |
| 11. (policy or consultation).mp. [mp=title, other title, abstract, heading words] |  |
| 12. 11 and 10 |  |
