# Supplementary material for: Non-medical prescribing in the United Kingdom National Health Service: A systematic policy review
Source: PLoS One. 2019 Jul 29;14(7):e0214630. doi: 10.1371/journal.pone.0214630 (PMC6663007; doi:10.1371/journal.pone.0214630)
Supplement: S1 Protocol — (PDF) [file pone.0214630.s003.pdf]

Systematic review

1. * Review title.

Give the working title of the review, for example the one used for obtaining funding. Ideally the title should state succinctly the interventions or exposures being reviewed and the associated health or social problems. Where appropriate, the title should use the PI(E)COS structure to contain information on the Participants, Intervention (or Exposure) and Comparison groups, the Outcomes to be measured and Study designs to be included.

Policy evolution and role of independent non-medical prescribing in the United Kingdom: a mixed methods systematic review

2. Original language title.

For reviews in languages other than English, this field should be used to enter the title in the language of the review. This will be displayed together with the English language title.

3. * Anticipated or actual start date.

Give the date when the systematic review commenced, or is expected to commence.

01/05/2015

4. * Anticipated completion date.

Give the date by which the review is expected to be completed.

31/01/2018

5. * Stage of review at time of this submission.

Indicate the stage of progress of the review by ticking the relevant Started and Completed boxes. Additional information may be added in the free text box provided.
Please note: Reviews that have progressed beyond the point of completing data extraction at the time of initial registration are not eligible for inclusion in PROSPERO. Should evidence of incorrect status and/or completion date being supplied at the time of submission come to light, the content of the PROSPERO record will be removed leaving only the title and named contact details and a statement that inaccuracies in the stage of the review date had been identified.
This field should be updated when any amendments are made to a published record and on completion and publication of the review. If this field was pre-populated from the initial screening questions then you are not able to edit it until the record is published.

The review has not yet started: No

Review stage	Started	Completed
Preliminary searches	Yes	Yes
Piloting of the study selection process	Yes	Yes
Formal screening of search results against eligibility criteria	Yes	Yes
Data extraction	Yes	Yes
Risk of bias (quality) assessment	Yes	Yes
Data analysis	Yes	Yes

Provide any other relevant information about the stage of the review here (e.g. Funded proposal, protocol not yet finalised).

Protocol finalised

Protocol finalised

6. * Named contact.

The named contact acts as the guarantor for the accuracy of the information presented in the register record.

Miss Graham-Clarke

Email salutation (e.g. "Dr Smith" or "Joanne") for correspondence:

7. * Named contact email.

Give the electronic mail address of the named contact.

emma.graham-clarke@nhs.net

8. Named contact address

Give the full postal address for the named contact.

Department of Anaesthetics, City Hospital, Sandwell and West Birmingham Hospitals NHS Trust, Dudley Road, Birmingham, B18 7QH

9. Named contact phone number.

Give the telephone number for the named contact, including international dialling code.

+44 (0)7971760603

10. * Organisational affiliation of the review.

Full title of the organisational affiliations for this review and website address if available. This field may be completed as 'None' if the review is not affiliated to any organisation.

School of Clinical and Experimental Medicine, University of Birmingham

Organisation web address:

www.birmingham.ac.uk

11. * Review team members and their organisational affiliations.

Give the title, first name, last name and the organisational affiliations of each member of the review team. Affiliation refers to groups or organisations to which review team members belong.

Miss Emma Graham-Clarke. School of Clinical and Experimental Medicine, University of Birmingham
Mr Timothy Noblet. School of Sport, Exercise and Rehabilitation Sciences, University of Birmingham
Professor John Marriott. School of Clinical and Experimental Medicine, University of Birmingham
Dr Alison Rushton. School of Clinical and Experimental Medicine, University of Birmingham

12. * Funding sources/sponsors.

Give details of the individuals, organizations, groups or other legal entities who take responsibility for initiating, managing, sponsoring and/or financing the review. Include any unique identification numbers assigned to the review by the individuals or bodies listed.

Self-funded PhD

13. * Conflicts of interest.

List any conditions that could lead to actual or perceived undue influence on judgements concerning the main topic investigated in the review.

None

14. Collaborators.

Give the name and affiliation of any individuals or organisations who are working on the review but who are not listed as review team members.

15. * Review question.

State the question(s) to be addressed by the review, clearly and precisely. Review questions may be specific or broad. It may be appropriate to break very broad questions down into a series of related more specific questions. Questions may be framed or refined using PI(E)COS where relevant.

1) What descriptors are there of the role of independent non-medical prescribing in the provision of healthcare? Research Aim: 1) To evaluate the literature concerning the use, facilitators and barriers of independent non-medical prescribing in primary and secondary care in the United Kingdom. Objective: 1) To conduct an extended systematic literature review to determine the position of independent non-medical prescribing, including review of facilitators and barriers that influence non-medical prescribers.

2) How does government policy view independent prescribing by non-medical practitioners, and has this changed since the initial introduction of independent prescribing by nurses and pharmacists? Research Aim: 2) To identify key policy documents supporting the use of independent non-medical prescribing and determine the current role of independent medical prescribing in the delivery of healthcare in the National Health Service. Objective: 2) To conduct a chronological review of government policy regarding the roles of independent non-medical prescribing in the provision of healthcare.

16. * Searches.

Give details of the sources to be searched, search dates (from and to), and any restrictions (e.g. language or publication period). The full search strategy is not required, but may be supplied as a link or attachment.

Objective 1

MEDLINE, CINAHL, EMBASE, AMED, BNI, ERIC, ASSIA, Web of Science, Open access theses and dissertations, Open grey, and SIGLE, will be searched for papers relating to the practice of independent non-medical prescribing. Papers that cite or are cited by the included papers will also be screened to identify any further relevant papers. All relevant papers will be included, Papers written in a language other than English will have their abstracts translated by an appropriate member of the university (staff or student). If the abstract indicates that the paper should be included in the analysis, then the full paper will be obtained and translated.

Objective 2

Database searches: Lexis Nexis, NHS evidence, UKOP, House of Commons Parliamentary Papers, HMIC, Web of Science, as well as the www.gov.uk (and national archives) website. A further search using Advanced Google search will also be conducted, using the key words, to capture any remaining relevant papers. The databases will be searched from the beginning of 2006 onwards.

Additional search strategy information can be found in the attached PDF document (link provided below).

17. URL to search strategy.

Give a link to a published pdf/word document detailing either the search strategy or an example of a search strategy for a specific database if available (including the keywords that will be used in the search strategies), or upload your search strategy.Do NOT provide links to your search results.

https://www.crd.york.ac.uk/PROSPEROFILES/19786_STRATEGY_20170101.pdf

Alternatively, upload your search strategy to CRD in pdf format. Please note that by doing so you are consenting to the file being made publicly accessible.

Yes I give permission for this file to be made publicly available

18. * Condition or domain being studied.

Give a short description of the disease, condition or healthcare domain being studied. This could include health and wellbeing outcomes.

Independent non-medical prescribing

Objective 1 - utilisation and uptake

Objective 2 - policy relating to the use of non-medical prescribing

19. * Participants/population.

Give summary criteria for the participants or populations being studied by the review. The preferred format includes details of both inclusion and exclusion criteria.

objective 1

Inclusion criteria:

Nurses, Allied health professionals, Physiotherapist, Pharmacist, Podiatrist, Chiropodist, Therapist

Exclusion criteria:

Doctor, Physician, Medical practitioner

Objective 2

Inclusion criteria:

policy relating to independent non-medical prescribing

Exclusion criteria:

policy relating to supplementary/dependent non-medical prescribing

20. * Intervention(s), exposure(s).

Give full and clear descriptions or definitions of the nature of the interventions or the exposures to be reviewed.

Objective 1

Inclusion criteria:

Independent non-medical prescribing

Exclusion criteria:

Supplementary prescribing, Dependent prescribing, Independent medical prescribing

Objective 2

as above, except papers that include both independent and supplementary prescribing will be included.

21. * Comparator(s)/control.

Where relevant, give details of the alternatives against which the main subject/topic of the review will be compared (e.g. another intervention or a non-exposed control group). The preferred format includes details of both inclusion and exclusion criteria.

Not applicable.

22. * Types of study to be included.

Give details of the types of study (study designs) eligible for inclusion in the review. If there are no restrictions on the types of study design eligible for inclusion, or certain study types are excluded, this should be stated. The preferred format includes details of both inclusion and exclusion criteria.

Objective 1Inclusion criteria :Qualitative and mixed-method research trials and studies relating to independent non-medical prescribingExclusion criteria:Narrative reports describing a service, opinion papers and case studies Objective 2Inclusion criteria:Both white and green papers will be included as well other relevant policy statements, consultation documents and reports that relate to the United Kingdom.Exclusion criteria:Policy and reports that relate to countries outside the United Kingdom

23. Context.

Give summary details of the setting and other relevant characteristics which help define the inclusion or exclusion criteria.

The review will include studies and papers relating to the United Kingdom only, in both primary and secondary care.

Studies relating to countries outside the United Kingdom will be excluded.

24. * Main outcome(s).

Give the pre-specified main (most important) outcomes of the review, including details of how the outcome is defined and measured and when these measurement are made, if these are part of the review inclusion criteria.

Objective 1

Facilitators, Barriers, Attitudes, Utilisation

Objective 2

the use of independent non-medical prescribing to improve healthcare provision.

Timing and effect measures

Not applicable.

25. * Additional outcome(s).

List the pre-specified additional outcomes of the review, with a similar level of detail to that required for main outcomes. Where there are no additional outcomes please state 'None' or 'Not applicable' as appropriate to the review

Objective 1

Secondary themes relating to more specific aspects may be able to be identified during the data extraction phase such as effect of training

Objective 2

N/a

Timing and effect measures

Not applicable.

26. * Data extraction (selection and coding).

Give the procedure for selecting studies for the review and extracting data, including the number of researchers involved and how discrepancies will be resolved. List the data to be extracted.

Both Objectives:

Initial screening of titles/abstracts obtained from all searches will be conducted to remove duplicates and the number removed will be recorded. The remaining abstracts and titles will be reviewed by two independent reviewers and any obviously unsuitable papers will be excluded at this stage and the numbers recorded. When studies appear to meet the eligibility criteria or when a decision cannot be made based solely on the title or abstract, full-text copies will be obtained, and two independent reviewers will assess the papers for eligibility for inclusion. If there is any disagreement between the two reviewers, then a third reviewer will be asked to mediate. Full details of the number of papers included and excluded at each stage will be recorded. Inter-rater reliability will be assessed using Cohen's kappa statistic. Data extraction for the policy documents

will use a standardised pre-formatted and pre-piloted form, with two reviewers extracting the data independently. Each reviewer will extract the data independently. The data extraction forms will be compared and any discrepancies identified. If it is not possible to resolve the discrepancies through discussion, then a third reviewer will be asked to mediate.

Objective 1

Data items to be extracted: Aims/Research Question, Ethics, Study design/Theoretical approach, Source of funding, Participant numbers and demographics, Study setting, Study methodology, Data Collection, Data Analysis, Key outcomes, Key themes, Key findings, Recommendations

Objective 2

Data items to be extracted: Date, Advisory, consultative or implementation, Context eg DH, advisory body, charity, Key messages, Potential sources of bias

27. * Risk of bias (quality) assessment.

State whether and how risk of bias will be assessed (including the number of researchers involved and how discrepancies will be resolved), how the quality of individual studies will be assessed, and whether and how this will influence the planned synthesis.

Objective 1

Two independent reviewers will assess risk of bias using the QATSDD tool and the results will be compared. If there is disagreement between the reviewers, which is unable to be resolved through discussion, then a third reviewer will be asked to mediate. Lower quality studies will be included in the synthesis, but reference will be made in the synthesis to the lower quality (with specific indications, such as lack of clarity over study design details). The risk of bias tool will be piloted beforehand to ensure familiarity with it.

Objective 2

Risk of Bias assessment is not applicable, however, potential sources of bias, such as documents produced by a single professional body, which may be inclined towards that profession, will be identified at the data extraction stage.

28. * Strategy for data synthesis.

Give the planned general approach to synthesis, e.g. whether aggregate or individual participant data will be used and whether a quantitative or narrative (descriptive) synthesis is planned. It is acceptable to state that a quantitative synthesis will be used if the included studies are sufficiently homogenous.

Objective 1

The intention is to conduct a thematic synthesis of the data. The papers will undergo thematic coding and analysis to identify recurrent themes, which then permits summarisation of the findings under descriptive theme headings. The findings will be presented in a tabular form, grouped according to the major themes identified. The absence of any supporting literature for any of the themes identified will be highlighted in the discussion of the final review.

In addition, a simple time line representation of the selected studies will be completed, to include date of publication and major findings.

Objective 2

A narrative synthesis of the data will be undertaken. The papers (and main findings) will be presented visually in the form of a timeline.

29. * Analysis of subgroups or subsets.

Give details of any plans for the separate presentation, exploration or analysis of different types of participants (e.g. by age, disease status, ethnicity, socioeconomic status, presence or absence or co-morbidities); different types of intervention (e.g. drug dose, presence or absence of particular components of intervention); different settings (e.g. country, acute or primary care sector, professional or family care); or different types of study (e.g. randomised or non-randomised).

None planned

30. * Type and method of review.

Select the type of review and the review method from the lists below. Select the health area(s) of interest for your review.

Type of review

Cost effectiveness
No

Diagnostic
No

Epidemiologic
No

Individual patient data (IPD) meta-analysis
No

Intervention
No

Meta-analysis
No

Methodology
No

Narrative synthesis
No

Network meta-analysis
No

Pre-clinical
No

Prevention
No

Prognostic
No

Prospective meta-analysis (PMA)
No

Review of reviews
No

Service delivery

No

Synthesis of qualitative studies
No

Systematic review
Yes

Other
No

Health area of the review

Alcohol/substance misuse/abuse
No

Blood and immune system
No

Cancer
No

Cardiovascular
No

Care of the elderly
No

Child health
No

Complementary therapies
No

Crime and justice
No

Dental
No

Digestive system
No

Ear, nose and throat
No

Education
No

Endocrine and metabolic disorders
No

Eye disorders
No

General interest
No

Genetics
No

Health inequalities/health equity
No

Infections and infestations
No

International development
No

Mental health and behavioural conditions
No

Musculoskeletal

No

Neurological
No

Nursing
No

Obstetrics and gynaecology
No

Oral health
No

Palliative care
No

Perioperative care
No

Physiotherapy
No

Pregnancy and childbirth
No

Public health (including social determinants of health)
No

Rehabilitation
No

Respiratory disorders
No

Service delivery
No

Skin disorders
No

Social care
No

Surgery
No

Tropical Medicine
No

Urological
No

Wounds, injuries and accidents
No

Violence and abuse
No

31. Language.

Select each language individually to add it to the list below, use the bin icon to remove any added in error.
English

There is an English language summary.

32. Country.

Select the country in which the review is being carried out from the drop down list. For multi-national collaborations select all the countries involved.

England

33. Other registration details.

Give the name of any organisation where the systematic review title or protocol is registered (such as with The Campbell Collaboration, or The Joanna Briggs Institute) together with any unique identification number assigned. (N.B. Registration details for Cochrane protocols will be automatically entered). If extracted data will be stored and made available through a repository such as the Systematic Review Data Repository (SRDR), details and a link should be included here. If none, leave blank.

Not applicable.

34. Reference and/or URL for published protocol.

Give the citation and link for the published protocol, if there is one

Give the link to the published protocol.

Alternatively, upload your published protocol to CRD in pdf format. Please note that by doing so you are consenting to the file being made publicly accessible.

Yes I give permission for this file to be made publicly available

Please note that the information required in the PROSPERO registration form must be completed in full even if access to a protocol is given.

35. Dissemination plans.

Give brief details of plans for communicating essential messages from the review to the appropriate audiences.

It is intended to submit the completed review to an appropriate peer-reviewed open-access healthcare journal for publication, and to present the findings at a suitable conference. It is also intended that the review will comprise part of a Ph.D. thesis, which will be available through the University of Birmingham library.

Do you intend to publish the review on completion?

Yes

36. Keywords.

Give words or phrases that best describe the review. Separate keywords with a semicolon or new line. Keywords will help users find the review in the Register (the words do not appear in the public record but are included in searches). Be as specific and precise as possible. Avoid acronyms and abbreviations unless these are in wide use.

Independent non-medical prescribing

facilitators

Barriers

Utilisation

Uptake

Policy

37. Details of any existing review of the same topic by the same authors.

Give details of earlier versions of the systematic review if an update of an existing review is being registered, including full bibliographic reference if possible.

National Institute for Health Research

38. * Current review status.

Review status should be updated when the review is completed and when it is published. For newregistrations the review must be Ongoing.

Please provide anticipated publication date

Review_Completed_not_published

39. Any additional information.

Provide any other information the review team feel is relevant to the registration of the review.

Objective one finished and published, objective two remains ongoing (11/6/18)

40. Details of final report/publication(s).

This field should be left empty until details of the completed review are available.

Graham-Clarke E, Rushton A, Noblet T, Marriott J (2018) Facilitators and barriers to non-medical prescribing

– A systematic review and thematic synthesis. PLoS ONE 13(4): e0196471.

https://doi.org/10.1371/journal.pone.0196471

Give the link to the published review.

http://journals.plos.org/plosone/article?id=10.1371/journal.pone.0196471
